# Therapy Targets SARS-CoV-2 Infection-Induced Cell Death

**DOI:** 10.3389/fimmu.2022.870216

**Published:** 2022-05-17

**Authors:** Zhoujie Zhu, Jiayi Shi, Long Li, Jinling Wang, Yufen Zhao, Huabin Ma

**Affiliations:** ^1^ Institute of Drug Discovery Technology, Ningbo University, Ningbo, China; ^2^ Qian Xuesen Collaborative Research Center of Astrochemistry and Space Life Sciences, Ningbo University, Ningbo, China; ^3^ School of Medicine, Xiamen University, Xiamen, China

**Keywords:** SARS-CoV-2, cell death, apoptosis, necroptosis, syncytia pyroptosis

## Abstract

Coronavirus Disease 2019 (COVID-19) caused by SARS-CoV-2 has become a global health issue. The clinical presentation of COVID-19 is highly variable, ranging from asymptomatic and mild disease to severe. However, the mechanisms for the high mortality induced by SARS-CoV-2 infection are still not well understood. Recent studies have indicated that the cytokine storm might play an essential role in the disease progression in patients with COVID-19, which is characterized by the uncontrolled release of cytokines and chemokines leading to acute respiratory distress syndrome (ARDS), multi-organ failure, and even death. Cell death, especially, inflammatory cell death, might be the initiation of a cytokine storm caused by SARS-CoV-2 infection. This review summarizes the forms of cell death caused by SARS-CoV-2 *in vivo* or *in vitro* and elaborates on the dedication of apoptosis, necroptosis, NETosis, pyroptosis of syncytia, and even SARS-CoV-2 E proteins forming channel induced cell death, providing insights into targets on the cell death pathway for the treatment of COVID-19.

## Introduction

**Figure 1:**
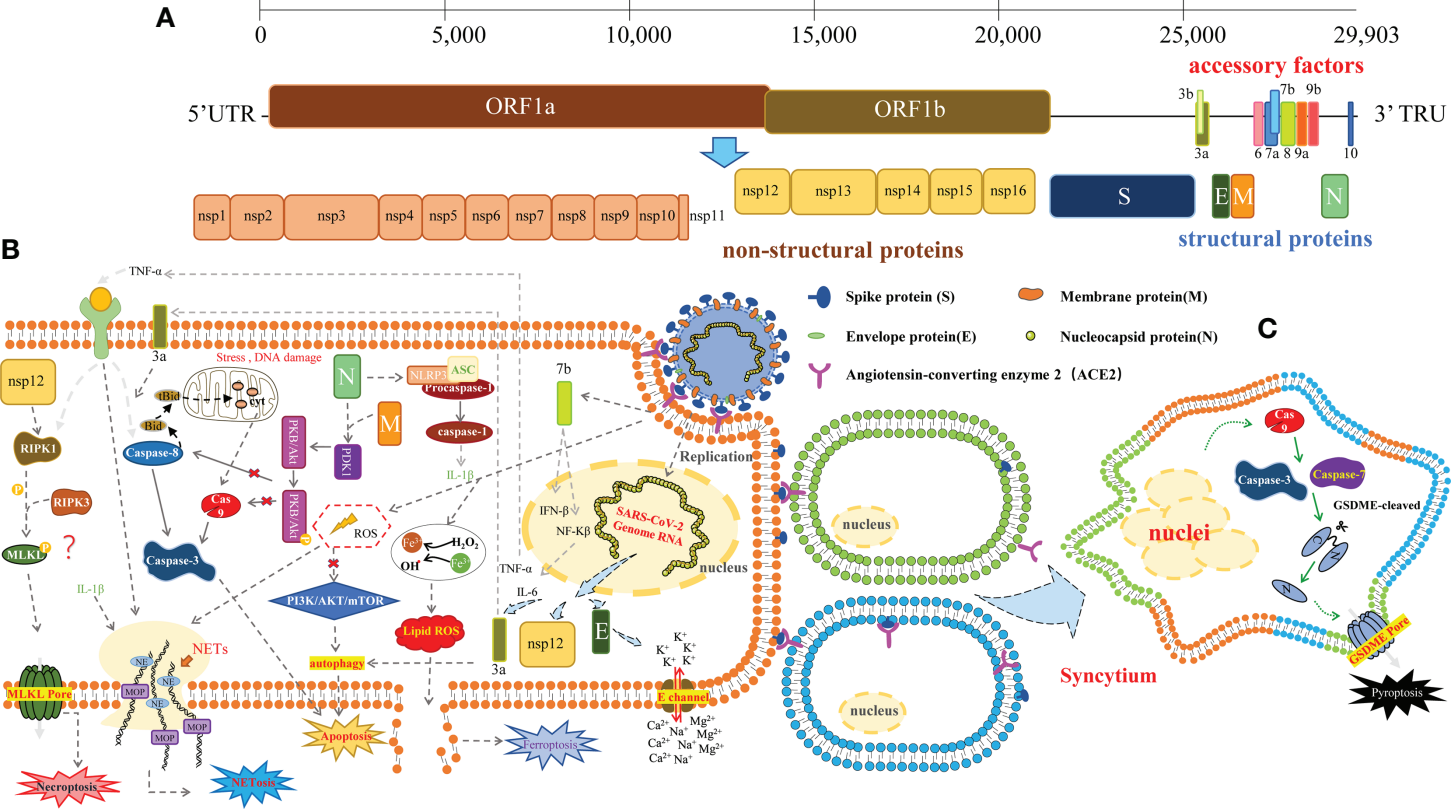
The schematic of SARS-CoV-2-induced cell death. **(A)** The SARS-CoV-2 virus particle and genome. The genome is a single-stranded RNA genome of which the full length is 29,903 bp. It includes ORF1a and ORF1b, which encode the 16 non-structural proteins, 9 accessory factors, and 4 structural proteins: spike protein (S), envelope protein (E), mbrane protein (M), and nucleocapsid protein (N). **(B)** SARS-CoV-2 induces various cellular stress: *apoptosis*, triggered by the extrinsic pathway (death receptor pathway), or the intrinsic pathway (mitochondrial pathway), involving the caspase cleavage. SARS-CoV-2 ORF3a caused apoptosis *via* the caspase-8/Bid extrinsic pathway; ORF7b can activate TNFα-induced apoptosis. Membrane (M) protein with nucleocapsid (N) protein *via* interacting with PDK1 and inhibiting the activation of PDK1-PKB/Akt signaling to trigger caspase-dependent apoptosis. Another structural protein spike of SARS-CoV-2 also induced autophagy and apoptosis by ROS-suppressed PI3K/AKT/mTOR signaling; *necroptosis*, mediated by RIPK1/RIPK3/MLKL. MLKL can be recruited by the autophosphorylated RIPK3 and subsequently phosphorylated by RIPK3 of human MLKL. Phosphorylated MLKL will form an MLKL pore, resulting in necroptosis. Nsp12 interacted with RIPK1 and activated it; *NLRP3 inflammasome*, consisting of NLRP3, ASC, and caspase-1, activated by N protein of SARS-CoV-2 interacted directly with NLRP3; SARS-CoV-2 E proteins form cation channels to trigger cell death independent of MLKL and gasdermins; NETosis, triggered by neutrophils and formed neutrophil extracellular traps (NETs) to release of chromatin structures containing myeloperoxidase and antimicrobial proteins to neutralize intruders. MPO, myeloperoxidase; NE, Neutrophil Elastase. *Ferroptosis*, triggered by iron accumulation and overload, or reactive oxygen species (ROS). **(C)** *Pyroptosis*, mediated by the gasdermin (GSDM) protein family. The N-terminal fragments of GSDM protein could induce the formation of membrane pores. SARS-CoV-2 S induces cell–cell fusion and syncytia formation driving caspase-9/GSDME-mediated syncytia pyroptosis.

Human coronaviruses (HCoVs) are known respiratory pathogens that could cause multiple respiratory diseases, ranging from the common cold and bronchitis to serious pneumonia (1, 2). Three of these viruses have been causing serious symptoms over the last years, including Severe Acute Respiratory Syndrome (SARS), Middle East Respiratory Syndrome Coronavirus (MERS), and now SARS Coronavirus 2 (SARS-CoV-2), especially SARS-CoV-2, which is responsible for the Coronavirus Disease 2019 (COVID-19) and has become a pandemic worldwide, causing millions of deaths and massive property losses (3–7). SARS-CoV-2 is a single-stranded RNA virus; belongs to the β-coronavirus; contains 29,903 nucleotides; encodes 16 non-structural proteins (NSP1–NSP16), 9 putative accessory factors, and 4 structural proteins, i.e., spike (S), envelope (E), membrane (M), and nucleocapsid (N); and spreads *via* respiratory droplets or close contact, which triggers mild or severe diseases (8–11) (**Figure 1A**). The main clinical symptoms of infected patients are cough, fever, and tachypnea; a CT scan usually reveals multiple patchy shadows. Severe infection can cause cytokine storms within the body, leading to multi-organ failure and even death (12–14). Cytokine storm is a life-threatening systemic inflammatory response syndrome that can be induced by pathogens, autoimmune disorders, or inflammatory cell death (15–18).

The autopsy of patients infected with SARS-CoV-2 is of great significance to truly understand the pathological changes of COVID-19 (19–27). Gupta et al. have well-reviewed that SARS-CoV-2 infection caused various injuries ranging from substantial respiratory to many extrapulmonary organs failure, including thrombotic complications, myocardial dysfunction and arrhythmia, acute coronary syndromes, acute kidney injury (AKI), gastrointestinal symptoms, hepatocellular injury, hyperglycemia and ketosis, neurologic illnesses, ocular symptoms, and dermatologic complications (28). Here, we mainly summarized how the different types of cell death caused by SARS-CoV-2 infection contribute to the organic failure directly, or indirectly, and discussed the therapy targets on the cell death signaling transduction molecules for treatment for COVID-19.

## Multiple Cell Death Pathways Were Induced in SARS-CoV-2 Infection

The cytopathic effect of cell death caused by the virus invading the host cells is a common result after the infection (29). Cell death in some instances can inhibit viral replication, but in more cases, it can enhance viral dissemination and affect the physiology of cells, leading to tissue and organ damage (30). The replication of coronaviruses in cells is regulated by many host factors, which can induce drastic structural and physiological changes in cells (2). During infection, SARS-CoV-2 could induce diverse cell death pathways (31, 32), such as apoptosis, necroptosis, pyroptosis, and NETosis in the host cells (**Figure 1B**).

Apoptosis is a major type of programmed cell death, morphologically characterized by cellular shrinkage, nuclear condensation, chromosomal DNA fragmentation, cytosolic membrane blebbing, and apoptotic body formation. It is triggered by the extrinsic (death receptor pathway) pathway, or the intrinsic (mitochondrial pathway) pathway, involving a group of cysteinyl aspartate proteases (caspases) cleavage (activation) (33–37).

SARS-CoV-2 infection can induce apoptosis *via* a variety of signaling pathways. It has been reported that the accessory protein ORF3a of SARS-CoV-1 caused cell death, vesicle formation, and Golgi fragmentation in VERO cells (38). To survey whether SARS-CoV-2 ORF3a can induce apoptosis, Ren and colleagues (39) overexpressed SARS-CoV-2 ORF3a in cultured HEK293T, HepG2, and VERO E6 cells; then stained the cells by annexin V-fluorescein 5-isothiocyanate (FITC)/propidium iodide (PI); and analyzed the apoptotic cells by flow cytometry. They found that SARS-CoV-2 ORF3a caused apoptosis *via* the caspase-8/Bid extrinsic pathway, which can be restored by z-VAD-fmk, a pan-caspase inhibitor. Importantly, SARS-CoV-2 ORF3a showed weaker proapoptotic activity than SARS-CoV-1 ORF3a in cultured cells, which might lead the virus to spread more widely. Consistently, two more groups demonstrated that SARS-CoV-2 ORF3a inhibited autophagic flux by blocking the fusion of autophagosomes/amphisomes with lysosomes, causing lysosomal destruction, which allowed the virus to escape the degradation by lysosomal (40, 41). These studies facilitated strategies targeting SARS-CoV-2 ORF3a or autophagic pathway for conferring potential protection against the spread of SARS-CoV-2. In support of this concept, a study by Gassen and colleagues demonstrated that targeting autophagic pathways on the polyamine pathway, and the control of BECN1 abundance through AKT1/SKP2 signaling by exogenous administration of spermidine and spermine, the selective AKT1 inhibitor MK-2206, and the BECN1-stabilizing anthelmintic drug niclosamide inhibited SARS-CoV-2 propagation *in vitro* and *in vivo* (42). Thus, both MK-2206 and niclosamide might be promising candidates for clinical trials.

ORF7b is another accessory protein of SARS-CoV-2, which can induce the transcription of IFN-β, TNF-α, and IL-6, activating type-I IFN signaling through IRF3 phosphorylation and activating TNFα-induced apoptosis in HEK293T cells and VERO E6 cells (43).

The membrane glycoprotein M of SARS-CoV-2 could trigger caspase-dependent apoptosis with the assistance of the nucleocapsid (N) protein *via* interacting with PDK1 and inhibiting the activation of PDK1-PKB/Akt signaling. Disruption of the M–N interaction by certain rationally designed peptides, abolished M-induced apoptosis, shedding light on a new aspect of drug designs on M–N interaction to prevent SARS-CoV-2 infection, which caused apoptosis (44).

Another structural protein spike of SARS-CoV-2 also induced autophagy and apoptosis in human bronchial epithelial and microvascular endothelial cells by reactive oxygen species (ROS)-suppressed PI3K/AKT/mTOR signaling, which then led to inflammatory responses, raising important implications for developing anti-inflammatory therapies, such as ROS and autophagy inhibitors, for COVID-19 patients (45).

In addition, a clinical report showed that a total of 17 of the 18 patients who died of COVID-19 suffered from lymphocytopenia, which is the main feature of severe COVID-19 disease (46). TUNEL staining showed that spleens and hilar lymph nodes (LNs) exhibited many lymphocyte apoptosis processes, which were caused by SARS-CoV-2 promoting Fas-mediated apoptosis of T and B lymphocytes.

Further, elevated serum levels of creatinine, tubular necrosis, and renal inflammation were observed in critically ill COVID-19 patients, consistent with AKI symptoms (47–49). To identify and uncover mechanisms specifically related to a SARS-CoV-2 protein that can induce cell death in AKI after SARS-CoV-2 infection, the SARS-CoV-2 nucleocapsid (N) structural protein-expressing plasmid was delivered into the normal mouse kidneys using a well-established non-invasive ultrasound-microbubble technique, which can induce AKI and exacerbate AKI under ischemic stress conditions. The mechanism lies in SARS-CoV-2 N interacting with Smad3 and enhances TGF-β/Smad3 signaling to arrest the G1 cell cycle leading to renal tubular epithelial cell apoptosis as labeled by TUNEL-positive cells. Moreover, both deletion of Smad3 and treatment with SIS3, the inhibitor of Smad3, can restore the SARS-CoV-2 N-induced AKI, which indicated that targeting Smad3 may represent a novel therapy for COVID-19-associated AKI (50).

Although we have summarized the apoptosis caused *via* different mechanisms induced by SARS-CoV-2, the underlying mechanisms of the massive inflammatory responses triggered by SARS-CoV-2 are largely limited. In contrast to necrosis, apoptosis is a form of clear cell death because the apoptotic bodies can be cleared through the phagocytic pathway by neighboring cells, without the release of cellular contents (51). We wonder whether inflammatory cell death occurred during SARS-CoV-2 infection. Indeed, analysis of the postmortem lung sections of fatal COVID-19 patients revealed that not only apoptosis but also necroptosis occurred in the lung, and the necrotic cell debris promoted massive inflammatory cell infiltration leading to lung damage in COVID-19 patients (52).

Necroptosis is an inflammatory type of programmed cell death mediated by RIPK1/RIPK3/MLKL. The occurrence of programmed necrosis could induce a series of morphological alterations in cells: with slight changes in the ultrastructure of the nucleus (especially the expansion of the nuclear membrane and the formation of small, irregular, and circumscribed patches by chromatin condensation), with increasing lucent cytoplasm and swelling organelles, the increased permeability of the cell membrane causes the cell to grow in size, resulting in the cell rupturing and the outflow of intracellular contents and provoking the inflammatory response of the surrounding tissues (53, 54). Mixed-lineage kinase domain-like (MLKL) is the main effector protein in necroptosis, which contains an N-terminal coiled-coil domain and a C-terminal kinase-like domain. MLKL can be recruited by the autophosphorylated RIPK3 and subsequently phosphorylated by RIPK3 at the threonine 357 and serine 358 residues of human MLKL (serine at positions 345, 347, and 352 and threonine at position 349 for mouse MLKL) (55–57). Phosphorylated MLKL will oligomerize and traffic to the plasma membrane, forming an MLKL pore, resulting in necroptosis (58).

As an important mediator of inflammation and cell death, RIPK1 can mediate the activation of caspase-8 to promote apoptosis or promote necroptosis by activating RIPK3 and MLKL (59–62). Based on some evidence of RIPK1 activation found in COVID-19 (63–65), Xu et al. used the lung pathological samples of COVID-19 patients and cultured human lung organoids and ACE2 transgenic mice infected by SARS-CoV-2 to explore the role of RIPK1 in SARS-CoV-2 infection. Although autopsy detection revealed that the expression of its downstream signaling molecule RIPK3 was found to be very low, and phosphorylated RIPK3 and MLKL were also undetectable, they found that the RNA-dependent RNA polymerase of SARS-CoV-2, NSP12, directly interacted with RIPK1 to promote its activation, resulting in the transcriptional induction of proinflammatory cytokines and host factors including ACE2 and EGFR, which promote viral entry into cells (66). As multiple RIPK1 inhibitors (Nec-1s, GSK′481/GSK′772, etc.) have been advanced beyond Phase I safety studies in human clinical trials (67, 68), the authors suggested that the RIPK1 kinase inhibitors may provide effective therapy for severe COVID-19.

During SARS-CoV-2 infection, the inhibitor of necroptosis did not completely block IL-1β secretion, suggesting that there may be other pathways involved in the inflammatory responses such as pyroptosis (52).

Pyroptosis is a lytic and inflammatory type of programmed cell death, which is characterized by the swelling of cells, forming a big balloon on the plasma membrane, destructing the cell plasma membrane, releasing the cellular contents, and causing lysis of cells (69, 70). This type of cell death is mediated by the gasdermin (GSDM) protein family (71), which is activated and cleaved by caspase protein or other proteases (72–79). The N-terminal fragments of GSDM protein could induce the formation of membrane pores, disrupting the cell membrane and causing eventual lysis (80, 81).

The participation of the inflammasome in COVID-19 has been highly speculated as to its main contribution to excessive inflammatory responses upon SARS-CoV-2 infection (82, 83). The NLRP3 inflammasome, consisting of NLRP3, ASC, and caspase-1, is activated in response to SARS-CoV-2 infection and is active in COVID-19 patients, which is associated with the clinical outcome of the disease (84). Furthermore, Pan and colleagues found that the N protein of SARS-CoV-2 interacted directly with NLRP3, promoted the recruitment of ASC, and facilitated NLRP3 inflammasome assembly, which resulted in the maturation of proinflammatory cytokines and triggered proinflammatory responses in cultured HEK293T or A549 cells. Notably, treatment with MCC950 (a specific inhibitor of NLRP3) and Ac-YVAD-cmk (an inhibitor of caspase-1) or genetic deletion of Nlrp3 inhibited N protein-induced lung injury and cytokine production (85). However, in cultured Calu-3 cells, the inhibitors of caspase-1 and NLRP3 had no effects on the production of IL-1β induced by SARS-CoV-2 infection but blocks caspase-8 using the inhibitor, or siRNA knockdown decreased the production and secretion of IL-1β (52). In this scenario, it is important to further determine specific mechanisms by which SARS-CoV-2 triggers the inflammasome activation and investigate which specific inflammasome platforms are activated during the disease for effective therapeutic strategies to target COVID-19.

NETosis, a form of regulated neutrophil death, is characterized by the formation and release of neutrophil extracellular traps (NETs), which are networks of myriad pathogen-associated molecular patterns (PAMPs), consisting of extracellular fibers composed of DNA containing histones and granule-derived enzymes (such as lactoferrin, cathepsins, neutrophil elastase (NE), and myeloperoxidase (MPO)), as well as cytoplasmic and cytoskeletal proteins. In addition to the NADPH oxidase (NOX)/ROS-, peptidylarginine deiminase 4 (PADI4)-, and NE-dependent pathways on the activation of NETosis, RIPK3/MLKL-mediated necroptosis and GSDMD-driven pyroptosis linked the excessive inflammatory response to NETosis (86–89). Emerging evidence from the clinic severe cases of COVID-19 implicated that NETosis and NET formation/release played a central role in the pathophysiology of inflammation, coagulopathy, immunothrombosis, and even organ damage during SARS-CoV-2 infection (90–94). With the growing roles of NETosis and NETs in COVID-19 reported, targeting dysregulated NETosis and NET formation/release is a new aspect of severe COVID-19 treatment. NETosis inhibitors (fostamatinib targeting SYK, etc.), or NET degraders (GSK 484 targeting PAD 4, Dornase alfa degrading cfDNA) were used in preclinical or clinical development as anti-COVID-19 drugs, which was well-summarized by other groups (93, 95–97). Here, we emphasized the Food and Drug Administration (FDA)-approved alcoholism-averting drug, disulfiram, which was identified as an inhibitor of GSDMD pore formation by covalently modifying human/mouse Cys191/Cys192 in GSDMD and preventing IL-1β release and pyroptosis (98). Although the linkage of GSDMD-mediated pyroptosis with NETosis has been reported (99, 100), Egeblad and colleagues recently found that treatment with disulfiram reduced NET formation, as well as lung inflammation and perivascular fibrosis in a golden hamster SARS-CoV-2 infection model *via* downregulated innate immune and complement/coagulation pathways (101).

## SARS-CoV-2 S Induced Cell–Cell Fusion and Syncytia Death

Cell fusion between eukaryotic cells is a common phenomenon, caused by various pathogens, including bacteria, parasites, and viruses, which involves a broad range of physiological and pathological processes (102). The virus-mediated cell–cell fusion will lead to the fusion of cell membrane and cytoplasmic contents between cells, forming the multinucleated giant cells, also known as syncytia. SARS-CoV-2 infection can induce cell–cell fusion and syncytia formation, which has been widely confirmed in the lungs and other tissues of infected patients (103–105), or *in vitro* cell culture systems (106–108), which was well-summarized by Schwartz and colleagues (109). Syncytia formation was mediated by cell–cell fusion occurring between the surfaces of cell membranes. Within the syncytia, cellular contents from different cells mixed and interacted, triggering various cellular responses. We aimed to discuss the fate determination of syncytia and its role in COVID-19 progress, providing insights into targeting syncytia death on COVID-19 treatment.

Recent works reported that both DNA damage response and cGAS-STING signaling pathway were activated upon cell–cell fusion, which was important for host antiviral responses (110, 111). Furthermore, Zhang and colleagues demonstrated that the multinucleate syncytia formed by SARS-CoV-2 infection could internalize multiple lines of lymphocytes to form typical cell-in-cell structures, remarkably leading to the death of internalized cells (112). Moreover, we found that syncytia formed by HeLa–spike cell fusion with HeLa-ACE2 cells died in parallel with the increased activity of caspase-3/7/9 and the cleavage of GSDME (108). Interestingly, the deletion of caspase-9 not only blocked the cleavage of GSDME and cell death but also abolished the S2′ fragment of SARS-CoV-2-S-Flag induced by cell–cell fusion, indicating a linkage between caspase-9 and SARS-CoV-2 S protein cleavage. Thus, targeting caspase-9 might be a promising strategy to prevent syncytia cell death (**Figure 1C**). To extend the pathophysiological role of this caspase-9/GSDME-mediated syncytia pyroptosis, single-cell RNA-sequencing (scRNA-Seq) data from eight normal human lung transplant donors with a total of 42,225 cells were analyzed, showing that both ACE2 and GSDME were expressed in AT2 cells in the human lung. Finally, we proposed that this lytic pyroptosis of syncytia may contribute to the excessive inflammatory responses in severe COVID-19 patients. In line with this idea, treatment with caspase-9 selective inhibitor, z-LEHD-fmk, markedly reduced SARS-CoV-2-induced lung damage in K18-hACE2 transgenic mouse model, which was evidenced by the reduced hemorrhage and inflammatory cell infiltration, as well as the alleviated proinflammatory response in the lung (113), while the authors demonstrated that this effect was due to intrinsic apoptosis inhibition by z-LEHD-fmk. Whether apoptosis switched to pyroptosis needs further investigation.

## SARS-CoV-2 E Proteins Form Cation Channels to Trigger Cell Death

Interestingly, consistent with the executors of pyroptosis (GSDMs) or necroptosis (p-MLKL) destroying the membrane integrity by forming either pores or channels, the envelope (E) protein, another structural protein of SARS-CoV-2, can form a cation channel to induce rapid cell death in myriad susceptible cell types and robust secretion of cytokines and chemokines in macrophages resulting in acute respiratory distress syndrome (ARDS)-like damages *in vitro* and *in vivo* (**Figure 1B**). Using a planar lipid bilayer recording system, the authors found that BE-12 (berbamine), a type of bisbenzylisoquinoline alkaloid, might be a candidate inhibitor for 2-E channels. Furthermore, to improve the antiviral activity, four more channel inhibitors (BE-30~33) were designed and synthesized based on BE-12. Finally, a new class of 2-E channel inhibitor BE-33 was identified, which exhibited not only high efficiency for antiviral activity both *in vitro* and *in vivo* but also negligible cytotoxicity, raising a promising antiviral strategy targeting 2-E channel (114). To discover SARS-CoV-2-E channel inhibitors, Wang and coworkers developed a cell-based high-throughput screening (HTS) assay and screened 4,376 compounds. Proanthocyanidins, a natural product widely used in cosmetics, were identified (115).

## Discussion and Perspectives

The abovementioned different types of cell death induced by SARS-CoV-2 infection have been demonstrated in all kinds of cells including epithelial cells, macrophages, and neutrophils. It was reported that ACE2-mediated SARS-CoV-2 spike infection could induce inflammatory responses and apoptosis of human bronchial epithelial and microvascular endothelial cells *via* enhancing autophagy, which might result in organ dysfunction (45). It was also found that SARS-CoV-2 N protein-mediated AKI may be caused by tubular epithelial cell apoptosis through the TGF/Smad3 signaling-dependent G1 cell cycle arrest (50). Using gene expression profiling, Jha et al. revealed that the apoptosis signaling pathway was activated in SARS-CoV-2-infected human lung epithelial cells, which may lead to cardiovascular complications of COVID-19 (116). A recent study showed that the non-structural protein 6 (NSP6) of SARS-CoV-2 could induce NLRP3-dependent pyroptosis in lung epithelial cells *via* binding to the vacuolar ATPase proton pump component ATP6AP1, while pharmacological rectification of autophagic flux by 1α,25-dihydroxyvitamin D3, metformin, or polydatin could be a novel therapeutic strategy to reduce pyroptosis in lung epithelial cells and improve clinical outcomes of COVID-19 (117). T- and B-lymphocyte apoptosis was also observed after SARS-CoV-2 infection, which may be the cause of lymphopenia, a common symptom in severe COVID-19 patients (46, 118). Aside from apoptosis, SARS-CoV-2-induced lymphocyte loss may also be due to cell–cell fusion-mediated syncytia death, which could be a potential therapeutic target for antiviral therapy in patients with COVID-19 (112). It was found that apoptotic markers were increased in plasmacytoid dendritic cells (pDCs), a cell type that is specialized in antiviral immunity to produce abundant type I interferons (IFNs). Hence, the diminished pDCs in COVID-19 patients may be associated with increased cell apoptosis (119). Ongoing pyroptosis was also found in circulating monocytes from COVID-19 patients with increased caspase-1 activation and lytic death (120, 121). Abundant cleared caspase-3 positive macrophages have been found in the lungs of patients with COVID-19, indicating that apoptosis may mediate the death of macrophages in COVID-19 lung tissues (66). Furthermore, SARS-CoV-2 spike infection can upregulate caspase-3 and caspase-6 expression to induce apoptosis in THP-1-like macrophages, which is likely mediated by the increase of ROS and intracellular calcium release (122). The pathological investigation of a clinical study demonstrated that SARS-CoV-2 infection caused severe lung injury *via* cell pyroptosis in pneumocytes and apoptosis in endothelial cells (123). Additionally, SARS-CoV-2 infection-induced apoptosis of endothelial cells may also lead to endotheliitis in various tissues including the lung, heart, kidney, and liver (124). Moreover, SARS-CoV-2 can promote NET formation in neutrophils under a process called NETosis, a form of neutrophil death, leading to multi-organ damage during the pathogenesis of COVID-19. Indeed, increased concentration of NETs has been detected in circulating and lung-infiltrating neutrophils from COVID-19 patients. Mechanistically, SARS-CoV-2-induced release of NETs might be mediated by ACE2, serine protease, virus replication, and PAD-4 (92, 125).

**Figure 2:**
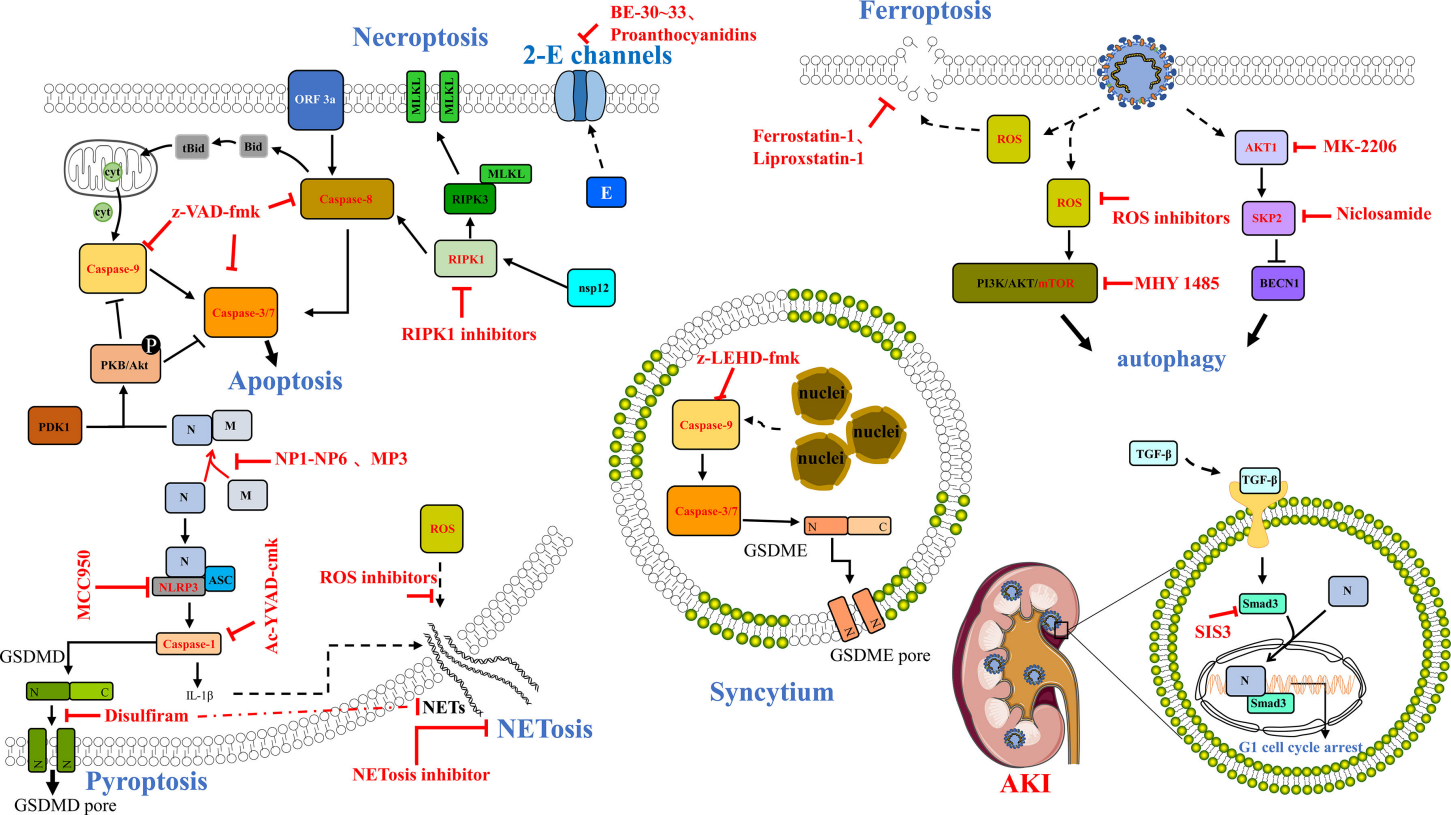
Potential compounds or drugs that targeted different cell death pathways. The potential therapeutics of drugs or compounds to inhibit cell death, including the use of z-VAD-fmk, a pan-caspase inhibitor, to reduce the caspase activity and block the apoptosis induced by SARS-CoV-2 ORF3a, and the use of RIPK1 inhibitors (Nec-1s, GSK′481/GSK′772, etc.), as well as using peptides such as NP1-NP6 and MP3 to disrupt the M–N interaction, and abolish the activity of N on the M-triggered apoptosis. In addition, a specific inhibitor of NLRP3 called MCC950 and an inhibitor of caspase-1 named Ac-YVAD-cmk can block NLRP3 inflammasome activation induced by SARS-CoV-2 N protein. Interestingly, disulfiram was revealed as an inhibitor of GSDMD that can effectively block pyroptosis and NET formation. BE-30~33 and proanthocyanidins could inhibit 2-E channel activity as channel inhibitors. Autophagy could be regulated using various treatments, such as the ROS inhibitor MHY1485, the AKT1 inhibitor MK-2206, and the BECN1-stabilizing anthelmintic drug/SKP2 inhibitor, niclosamide. Ferroptosis inhibitors including ferrostatin-1 and liproxstatin-1 might also be potential drug candidates for COVID-19. Further, z-LEHD-fmk, the caspase-9 selective inhibitor could suppress syncytium formation. SIS3, Smad3 pharmacological inhibitor, can inhibit SARS-CoV-2 N-induced AKI. The compounds or drugs are marked in bold and red.

SARS-CoV-2 infection can cause severe respiratory tract disease and lung injury and threaten human life, while there is still no special prevention or treatment at present. When the virus infects cells, many factors are involved in the pathogenesis of the host disease, leading to human death. In this review, we focused on the multiple types of cell death such as apoptosis, necroptosis, pyroptosis, NETosis, and other undefined death triggered by SARS-CoV-2 infection *in vivo* or *in vitro*; then we discussed the relationship between inflammatory cell death and cytokine storm, raising the possibility for targeting cell death pathway for the treatment of COVID-19 (**Figure 2**). Among these, we highlighted some potential compounds or drugs targeting the molecules of cell death pathway, such as RIPK1 (66), caspase-9 (108), Smad (50), SARS-CoV-2 Orf3a (39–41), and E protein (114). What is more, iron metabolism dysfunction has also been found in COVID-19 patients; for example, the serum ferritin levels were higher in severe COVID-19 patients than in mild cases, which may cause iron accumulation and overload, which trigger ferroptosis (126). Yang and Lai hypothesized that ferroptosis might serve as a new treatment target, and the improved ferrostatin-1 and liproxstatin-1 analogs might be potential drug candidates for COVID-19 (127). Interestingly, disulfiram, an alcoholism-averting drug approved by the FDA, was recommended to be a potential therapeutic target for SARS-CoV-2 infection in Phase 2 clinical trials by targeting SARS-CoV-2 main protease, 3CLpro (128–130). However, it has been identified that disulfiram is covalently targeted on human/mouse Cys191/Cys192 of GSDMD protein leading to blocking the GSDMD pore formation, IL-1β release, and pyroptosis (98). Furthermore, another study showed that disulfiram can inhibit the NET formation and protect rodents from SARS-CoV-2 infection (101). All these raise a common point that one drug/compound might target various proteins even on multiple signaling pathways to either synergically exert effects or trigger off-target toxicities. Therefore, we need to deeply explore the cell types of death caused by SARS-CoV-2 infection, reveal the molecular mechanism of cell death, and accurately regulate cell death by using specific pharmacological therapies to reduce the occurrence and prognosis of COVID-19.

Nevertheless, the impact of the damaged or dead cells on the injured tissues and organs is still not well understood. Furthermore, whether cytokines released by cell death participate in cytokine storms has not been well described yet. However, research on the mechanism of SARS-CoV-2 infection-induced cell death may provide additional perspectives for antiviral therapies and the development of anti-SARS-CoV-2 drugs.

## Author Contributions

LL, JW, YZ, and HM: conceptualization. ZZ and JS: investigation and writing—original draft preparation. LL, JW, and HM: writing—review and editing. All authors have read and agreed to the published version of the manuscript.

## Funding

This work was supported by the Natural Science Foundation of Zhejiang Province (No. LQ21H030002), the Scientific Research Grant of Ningbo University (215–432000282), and the Ningbo Top Talent Project (215–432094250).

## Conflict of Interest

The authors declare that the research was conducted in the absence of any commercial or financial relationships that could be construed as a potential conflict of interest.

## Publisher’s Note

All claims expressed in this article are solely those of the authors and do not necessarily represent those of their affiliated organizations, or those of the publisher, the editors and the reviewers. Any product that may be evaluated in this article, or claim that may be made by its manufacturer, is not guaranteed or endorsed by the publisher.
